# Characteristics of isolated multiresistant bacteria according germ, location and “zero resistance” criteria

**DOI:** 10.1186/2197-425X-3-S1-A134

**Published:** 2015-10-01

**Authors:** S Carvalho Brugger, R Gavilan Rabell, M Miralbes Torner, G Jimenez Jimenez, S Iglesias Moles, J Trujillano Cabello, M Vallverdú Vidal, B Balsera Garrido, F Barcenilla Gaite, M Palomar Martínez

**Affiliations:** Hospital Universitari Arnau de Vilanova, Lleida, Spain

## Introduction

Multiresistant bacteria (MRB) development is a growing phenomenon. In 2013, the “Zero Resistance” (RZ) program was launched in Spain, to help prevent the emergence of multiresistant bacteria (MRB) in critically ill patients. One of its recommendations is to complete a checklist upon patient admission in Intensive Care Unit (ICU) to identify those patients at high risk for colonization or infection by MRB*.

## Objectives

To search the relation between most common MRB and specific risk factors for colonization and infection, and the location of the samples where they are identified.

## Methods

A prospective study from March 17^th^, 2014 to January 31^rst^, 2015. All patients admitted to a polyvalent ICU of a general hospital were submitted to the checklist proposed, with the application of contact precaution (CP) measures in patients at risk for colonization or infection by MRB. Bacteriologic swabs (nasal, pharyngeal, axillary and rectal) were routinely performed to all patients admitted, besides diagnostic cultures when needed. Furthermore, we analysed other pathological variables and comorbidities. The difference between groups of MRB was made by Chi-square test for qualitative variables and the Kruskal-Walls test for the continuous ones. Statistical significance was set at p < .05.

## Results

In 78 patients were identified one or more MRB (in total 91 MRB). They were classified in 6 groups. 29 patients (37,2%) were MRSA carriers, 22 (28,2%) *E coli* ESBLs, 10 (21,8%) *Klebsiella spp* ESBLs, 10 (12,8%) *P aeruginosa*, 8 (10,2%) *Acinetobacter spp* and 12 (15,4%) others MRB carriers (including 1 case of carbapenemase). Nasal swabs detected 26 (33,3%) of MRB carriers [22 (75,9%) of all MRSA], pharyngeal swabs 26 (35,9%) [17 (43,6%) of MRSA], axillary swabs 13 (16,7%) [7 (24,1%) of MRSA, 3 (37,5%) of *Acinetobacter spp*], and rectal swabs 30 (38,5%) [27 (87,1%) of ESBLs, 3 (37,5%) of *Acinetobacterspp*]. Diagnostic cultures (blood, urine, bronchoaspirate, surgical wound and others) detected MRB in less than 30% of the cases. The checklist did not detected neither colonization nor infection by MRB in 29 (37,2%) patients (44,8% MRSA, 50% *Acinetobacter spp*, 38,7% of ESBLs). We did not find significant relation with other comorbidities.

## Conclusions

In our environment, we detected a predominance of MRSA, higher than the Spanish media, followed by ESBLs. MRSA carriers were identified mostly by nasal swabs while the ESBL carriers were identified mainly by rectal swabs. We did not find significant relation between BMR and other comorbidities, possibly because of the sample size. The RZ checklist did not identify almost 40% of the MRB.

## References

[1] Montero J (2015). (Scientific Expert Committee for the “Zero Resistance” Project). Combatting resistance in intensive care: the multimodal approach of the Spanish ICU “Zero Resistance” program. Critical Care.

